# Morphology-Dependent Transformation of Dicalcium Phosphate Dihydrate (DCPD) to Octacalcium Phosphate (OCP) and Its Stability in Simulated Physiological Fluids

**DOI:** 10.3390/molecules30173631

**Published:** 2025-09-05

**Authors:** Daniela Chávez-Herrera, Estefanía Rangel-Villanueva, Mercedes Salazar-Hernández, Alfonso Talavera-Lopez, Alba N. Ardila A., Rosa Hernández-Soto, Oscar Joaquín Solis-Marcial, Jose A. Hernández

**Affiliations:** 1 Unidad Profesional Interdisciplinaria de Ingeniería Campus Guanajuato, Instituto Politécnico Nacional, Guanajuato 36275, Mexico; dchavezh1700@alumno.ipn.mx (D.C.-H.); irangel1801@alumno.ipn.mx (E.R.-V.); rohernandezs@ipn.mx (R.H.-S.); 2 Departamento de Ingeniería en Minas, Metalurgia y Geología, División de Ingenierías, Universidad de Guanajuato, Guanajuato 36020, Mexico; merce@ugto.mx; 3 Unidad de Ciencias Químicas, Universidad de Zacatecas, Campus UAZ Siglo XXI, Carretera a Guadalajara Km 6, Ejido la Escondida, Zacatecas 98160, Mexico; talavera@uaz.edu.mx; 4 Facultad Ciencias Básicas, Sociales y Humanas, Politécnico Colombiano Jaime Isaza Cadavid, Carrera 48 No. 7-151, Medellin 051052, Colombia; anardila@elpoli.edu.co; 5 Unidad Profesional Interdisciplinaria de Ingeniería Campus Zacatecas, Instituto Politécnico Nacional, Calle Circuito del Gato 202, Zacatecas 98160, Mexico; ojsolis@ipn.mx

**Keywords:** octacalcium phosphate, brushite, simulated gastric fluid, simulated body fluid, apatites

## Abstract

Calcium phosphate (CaP) materials are biocompatible and non-toxic to the body. However, they lack biointegration, exhibit a low resorption rate and can cause fibrous encapsulation throughout the implant material. A promising approach for dental or orthopedic regeneration is the use of dicalcium phosphate dihydrate (DCPD) and octacalcium phosphate (OCP), as they are well-suited to bone components. From a novel perspective, these apatites can be used as drug carriers for individuals with low tolerance to common excipients. In this study, the transformation of DCPD into different morphologies in DMEM was investigated using an induced dissolution and reprecipitation reaction solution. The DCPD transformation time was observed to be morphology-dependent and can occur between 48 and 168 h. In the interaction with simulated body fluid (SBF), simulated gastric fluid (SGF) and a combination of both (BFS/SGF), a higher mass loss was observed in SGF (~80%) than in the other fluids (~35%). The structural changes presented in DCPD and OCP before and after immersion in physiological fluids were analyzed by ATR-FTIR, SEM, XRD and EDS. The obtained OCP showed low stability in SGF compared to SBF and SBF/SGF, which indicates that it may be a suitable candidate for drug delivery in the digestive tract.

## 1. Introduction

Each year, more than 2 million bone grafting procedures are performed worldwide. The graft consists of an inorganic phase of mineral components (phosphate, calcium and hydroxyl groups) and an organic phase composed of collagen, which, when combined, give the bone strength and flexibility [1,2,3]. The regeneration mechanism is impaired due to bone fractures caused by bone loss. Osteological substitutes with compounds that allow bone regeneration as mechanical support are being sought [4,5]. Among the most studied synthetic bone grafts are calcium phosphates, in the form of calcium phosphate cement (CaP), which include apatites [6,7,8]. These materials are among the most important compounds found in nature due to characteristics that are similar to those of the inorganic components of bones. They have excellent biocompatibility, high bioactivity and low toxicity [9,10]. These compounds have multiple applications in various fields, including medicine, since they are used as fillings for dental defects and bone replacement, making them good candidates for bone tissue engineering applications [4,9,10,11,12]. CaPs such as hydroxyapatite (HA, Ca_5_(PO_4_)_3_OH), brushite (DCPD, CaHPO_4_·2H_2_O) and tricalcium phosphates (TCP, Ca_3_(PO_4_)_2_)) are used as precursors for the preparation of bone bioceramics and cements. Bioceramics usually consist of apatites that have advantages over pure HA or β-TCP due to their controllable biodegradation and better bioactivity, making them favorable for bone defect repair due to their good osteoconductivity and bioactivity [13,14]. HA, which is a bioceramic that does not have any toxic effect on the body and has great biocompatibility, has been used for the release of poorly soluble drugs with limited bioavailability [15,16,17,18]. Another apatite that meets the same conditions as HA is DCPD, which has good bioactivity, bioabsorbability, biodegradability and biocompatibility, which allows it to be rapidly resorbed in vivo, followed by the formation of new bone tissues under physiological conditions [5,6,19,20].

DCPD is considered one of the most feasible precursors of biological apatites, along with amorphous calcium phosphate (ACP, Ca_2_H_y_(PO_4_)_x_·nH_2_O) and octacalcium phosphate (OCP, Ca_8_(HPO_4_)_2_(PO_4_)_4_·5H_2_O) [21,22]. OCP is an osteoconductor that stimulates bone cells and promotes bone regeneration processes due to the formation of new bone through osteoblasts and its biodegradation by osteoclast-like cells [23,24]. OCP can present various morphologies, such as prismatic crystals, needles or elongated crystals, granules or spherical particles, and sheets or plates, depending on the synthesis [2,3,10,20,23,24]. One method of OCP synthesis is through crystallization using Dulbecco’s modified Eagle medium (DMEM), which contains a combination of essential nutrients, minerals, vitamins and amino acids necessary to maintain cell growth and viability in culture [2,16,25,26]. There are several processes for obtaining OCP from DCPD in which the same pH conditions (7.0) and temperature (37 °C) are used with the precipitation method, using CaCl_2_ as a source of Ca^2+^ and pH-stat, where the nucleation process occurs in alkaline conditions [27,28]. Another method is where a simulated body fluid (SBF) with the presence of Ca^2+^ is used, in which DCPD is immersed, observing the transformation into OCP from 1 day to 6 months [23]. One method to synthesize OCP is hydrothermal transformation using Dulbecco’s modified Eagle medium (DMEM), in which it has been observed that only the presence of Ca^2+^ ions influences the transformation of DCPD to OCP [25,26,29]. Furthermore, it was noticed that the transformation time decreases from 72 h to 2 h when the temperature is increased from 36.5 °C to 80 °C without stirring [29]. Based on this, the transformation of DCPD with different morphologies to OCP crystals is proposed in the present study using DMEM with stirring at a temperature of 36.5 °C. DCPD and OCP are immersed in SBF and simulated gastric fluid (SGF), and the combination of both fluids allows us to analyze the factors that affect the stability and undesirable properties of apatites with different structures, allowing us to consider biomaterials as drug vehicles.

## 2. Results and Discussion

### 2.1. Synthesis and Analysis of DCPD and OCP Degradation by Immersion in Different Simulated Fluids

The synthesis of DCPD with WL and FP morphologies yielded 44.5 ± 0.32% and 49.0 ± 0.24%, respectively. These results can be influenced by several factors, including temperature, pH and the initial concentration of the reactants, which is consistent with reports in the literature [30], which indicates that the maximum expected yield is less than 50%. Before immersion in the simulated fluids, a triplicate test was carried out with only water and DCPD at a 1/10 ratio (weight/volume), with a mass recovery after drying of 95.1 ± 0.4% for DCPD-FP and 94 ± 0.2% for DCPD-WL. Immersion of DCPD-FP in different simulated fluids showed that degradation depended on the medium in which it was immersed, with mass losses of 33.0 ± 0.17%, 40.2 ± 0.12% and 47.1 ± 0.22% for SBF, SGF and SBF/SGF, respectively. In the case of DCPD-WL, degradation after immersion was 35.8 ± 0.18%, 49.7 ± 0.40% and 27.1 ± 0.21% for SBF, SGF and SBF/SGF, respectively. This degradation may be associated with pH conditions, DCPD morphology and the presence of ions in the different simulated fluids due to the reactions occurring on the material surface [31].

In the transformation of DCPD to OCP, yields of 69.4 ± 0.21% were determined for the WL morphology and 79.9 ± 0.56% for FP. These results demonstrate that DCPD morphology directly influences the transformation to OCP, along with immersion time in DMEM. The OCP-FP obtained, when immersed in the different physiological fluids, showed degradations of 32.2 ± 0.13%, 79.6 ± 0.72% and 42.5 ± 0.28% for SBF, SGF and SBF/SGF, respectively. In the case of OCP-WL, degradation after immersion was 48.1 ± 0.34%, 73.4 ± 0.66% and 45.5 ± 0.18% for SBF, SGF and SBF/SGF, respectively. These results indicate that, in SGF, the presence of enzymes and the pH (at 1.2) of the medium significantly affect the stability of the OCP obtained with both morphologies compared to SBF and SBF/SGF [30,32].

### 2.2. Attenuated Total Reflectance Fourier Transform Infrared (ATR-FTIR) Spectroscopy of Synthesized DCPD Before and After Immersion in Simulated Fluids

**Figure 1:**
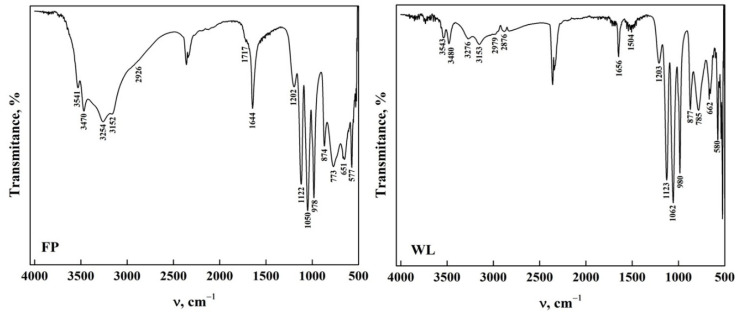
ATR-FTIR from DCPD in the form of WL and FP.

Figure 1 shows the ATR-FTIR spectra of DCPD with the two morphologies. Characteristic bands were observed in the stretching and bending mode of the H_2_O molecule in the 4000–1500 cm^−1^ region (functional group zone) [14,33,34,35]. It was noted that DCPD-FP has a higher intensity than DCPD-WL. In the fingerprint zone of the spectra (1500–400 cm^−1^), a similarity is observed in the bands found in both spectra, referring to the phosphate groups (HPO_4_^2−^ and PO_4_^3−^) and the stretching of P-O and P-OH [36,37,38,39]. It is also observed that DCPD-FP has a higher intensity and better definition of bands than DCPD-WL, indicating a greater presence of functional groups on the surface of the biomaterial [14,17,35,40]. This allows us to assume that the spectra obtained from synthesized DCPD-FP better present the main characteristics of apatite than those from DCPD-WL [33,35,36,37,38,39,40]. All the bands observed in the spectra and their assignment are shown in Table 1.

**Figure 2:**
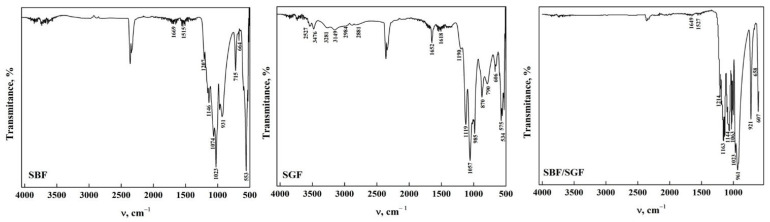
ATR-FTIR of DCPDWL form after its interaction with simulated fluids.

The ATR-FTIR spectra of DCPD-WL after immersion in different simulated fluids are shown in Figure 2. Significant changes in the stretching and bending zone of the O-H groups could be noticed, which are due to the nature of the solutions [20,33,41,42,43]. In DCPD immersed in SBF, it can be observed that only the weak bands at 3548, 3282, 2969 and 2866 cm^−1^ are present compared to DCPD before immersion. This may be due to the change in the concentration of the functional groups due to the components of the simulated fluid [23,33,34,44]. Nevertheless, when DCPD is immersed in SGF, most of the bands remain; nonetheless, their intensity decreased, and the position of the bands changed with respect to those found in DCPD before immersion. This indicates that the changes in the apatite structure are limited due to the composition of the simulated fluid [33,34,36,41]. In the immersion with the SBF/SGF combination, the bands from 4000 cm^−1^ to 1700 cm^−1^ disappear and the intensity of the different bands related to the functional groups decrease significantly in comparison with DCPD before their interaction [23,33,34,41]. In the fingerprint region of the ATR-FTIR spectra, changes in the intensity and position of the phosphate group bands were observed after immersion in the simulated fluids [17,32,33,35]. The appearance of bands around 1520 cm^−1^ and 1023 cm^−1^ corresponding to the O-H-O bending mode of the residual H_2_O and the stretching of the PO_3_^4−^ (ν_3_) group were also observed [35,36,37,38,43,44]. The immersion time in SBF of DCPD did not allow transformation to OCP to take place; as mentioned in the literature, it needs around 24 h or a temperature higher than 70 °C [17,29]. All the bands observed in the spectra are shown in Table 2.

**Figure 3:**
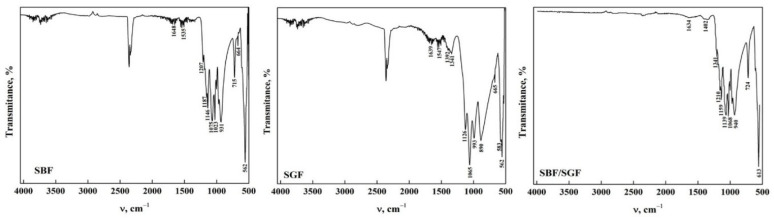
ATR-FTIR of DCPD-FP form after its interaction with simulated fluids.

The ATR-FTIR spectra of DCPD-FP after immersion in the simulated fluids are shown in Figure 3. It could be observed that the position of the bands after immersion in SBF is similar to that of synthesized DCPD, although there is a decrease in the intensity of some bands. In addition, a band at 1515 cm^−1^ was observed that corresponds to the O-H bending and rotation mode in the H_2_O molecule [34,35,42,43]. This indicates that apatite stability is affected by the concentration of ions present in the fluid [40,44]. In DCPD after immersion in SGF, a similar situation occurs with respect to immersion with SBF, where the bands decrease in intensity and change position. In addition, bands related to the bending and rotation of water molecules appear at 1575 cm^−1^ and 1402 cm^−1^, respectively [34,35,42,43]. Other bands at 1341 cm^−1^ and 1292 cm^−1^ are attributed to the P-O-H bending mode [34,35,36,37,38,41,43,44]. In the case of DCPD after immersion in SBF/SGF, all bands between 4000 cm^−1^ and 1700 cm^−1^ disappear, and a shoulder appears at 1341 cm^−1^ corresponding to the P-O-H bending mode [34,35,36,37,38,43,44]. The combination of both simulated fluids allows the changes in the ATR-FTIR spectra to take place in a more significant way compared to their individual effect. The immersion time in SBF and SBF/SGF (12 h) of DCPD did not allow the transformation of DCPD to OCP to take place; as mentioned in the literature, it needs around 24 h or a temperature higher than 70 °C [23,29]. The bands observed in the spectra and their assignment are shown in Table 3.

### 2.3. Attenuated Total Reflectance Fourier Transform Infrared (ATR-FTIR) Spectroscopy of Synthesized OCP Before and After Immersion in Simulated Fluids

**Figure 4:**
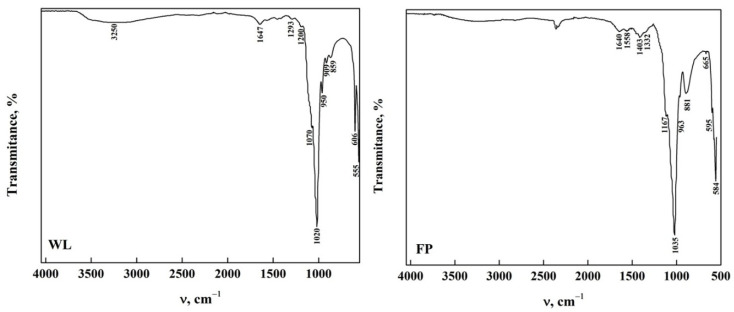
ATR-FTIR of OCP from DCPD (WL and FP).

The ATR-FTIR spectra of OCP obtained from the phase transformation reaction by dissolution–precipitation of DCPD immersed in DMEM for 168 h are observed in Figure 4. In the OCP-WL and OCP-FP obtained, it was observed that the bands in the functional group region disappeared, except for a wide band around 3254 cm^−1^ that corresponds to the stretching of the O-H extension in the H_2_O molecule [20,23,34,35,44]. These changes indicate that there is an alteration of some functional groups present on the DCPD surface that allow the transformation to OCP [8,20,34,44]. In the area between 1700 cm^−1^ and 400 cm^−1^, the appearance of bands at 1158 cm^−1^ and 1403 cm^−1^ is attributed to the bending mode and the CO_3_^2−^ (ν_3_) stretching vibrations [8,23,42]. Moreover, the band corresponding to the CO_3_^2−^ bending mode (ν_3_) was detected at 881 cm^−1^ [7,26,35,36,37,38,41]. The presence of carbonate and phosphate groups confirms the transformation of DCPD to OCP [23]. Figures S1 and S2 show the spectra of OCP-Wl and OCP-FP obtained at different immersion times in DMEM. It can be observed that the transformation of DCPD-WL is slow since OCP is obtained after 50 h of immersion in comparison with the transformation of DCPD-FP, where OCP-FP is obtained after 48 h. This may be influenced by the morphology, which could hinder a homogeneous transformation [29,35,40,43,45]. All the bands observed in the spectra and their assignment are shown in Table 4.

**Figure 5:**
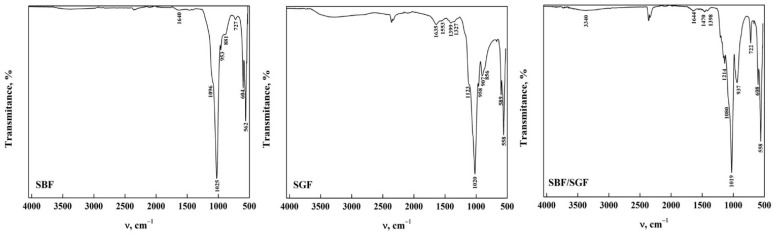
ATR-FTIR of OCP (WL) form after its interaction with simulated fluids.

The ATR-FTIR spectra of OCP-WL after immersion in the simulated fluids are shown in Figure 5. In case of interaction with SBF, it was observed that the bands in OCP between 4000 cm^−1^ and 1700 cm^−1^ disappear in SBF. This indicates that the HA particles that may form due to the change in pH during the interaction result in nucleation of HA crystals [22,35,43,46]. Furthermore, the presence of SBF may contribute to the transformation of apatite during the immersion time [17,23,29]. The presence of a broad band around 3276 cm^−1^ can be observed in OCP after immersion in SGF and SBF/SGF, indicating that the biomaterial may present a gradual transformation process caused by the low pH of the simulated fluid [22,47]. Furthermore, it was observed that the bands corresponding to the carbonate group significantly decrease their intensity, indicating a direct participation between the ions present in the simulated fluids and OCP [23,43]. This could be closely related to the morphology of the OCP obtained from DCPD, allowing us to infer that immersion in the simulated fluids is directly affected by the starting structure to obtain OCP-WL [48]. All the bands observed in the spectra and their assignment are shown in Table 5.

**Figure 6:**
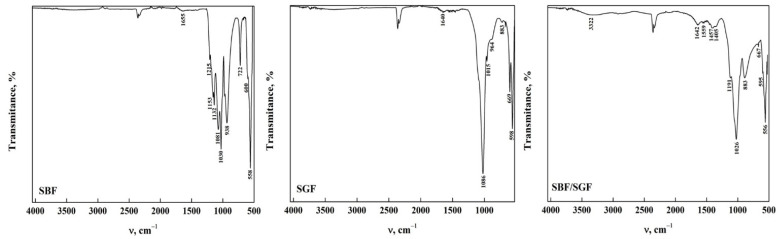
ATR-FTIR of OCP (FP) form after its interaction with simulated fluids.

Figure 6 presents the ATR-FTIR spectra of the OCP-FP after immersion in the simulated fluids. It is observed that the bands between 4000 cm^−1^ and 1700 cm^−1^ disappeared when the OCP was immersed in SBF and SGF, except for a broad band around 3302 cm^−1^ that remains after immersion. This is due to the formation of a layer in OCP when SBF was immersed and the degradation that occurred when SGF was immersed [30,31]. When immersed in SBF/SGF, it is observed that the O-H group band maintains an intensity that allows us to infer that the pH of the solution changes with the combination of both simulated fluids [36,41,43]. Most of the bands in the range between 1700 cm^−1^ and 400 cm^−1^ remain after immersion in the simulated fluids, including those corresponding to CO_3_^2−^ bending and stretching at around 1560 cm^−1^ and 1453 cm^−1^, respectively [23,35,43]. A decrease in the band assigned to the CO_3_^2−^ bending mode (ν_2_) was observed around 882 cm^−1^, indicating changes in the structure of the carbonate groups [19,35,43]. These results indicate that OCP can be used as an excipient due to its high degradation in SGF, which would allow release of the drug. All the bands observed in the spectra and their assignment are shown in Table 6.

### 2.4. Scanning Electron Microscopy (SEM) of Synthesized DCPD Before and After Immersion in Simulated Fluids

**Figure 7:**
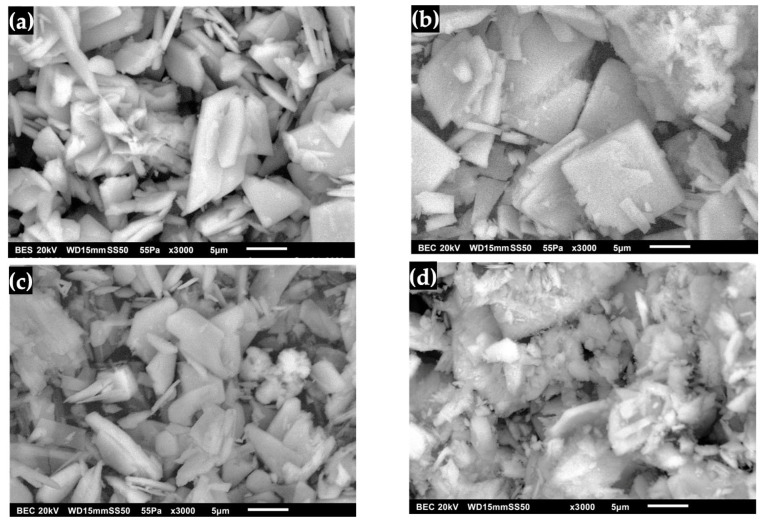
DCPD morphology with FP: (**a**) synthesized, (**b**) immersed in SBF, (**c**) immersed in SGF and (**d**) immersed in SBF/SGF.

The morphology of DCPD with an FP structure is shown in Figure 7a, where compacted powder-like plates can be observed growing rapidly in two directions with a microrectangular appearance on the surface [36,37,41,42,51]. Figure 7b shows the micrographs of DCPD after immersion in SBF. Significant changes in the surface can be noticed due to a better definition of square lamellar particles and rectangular amorphous crystals forming angles with each other, and large square particles are no longer observed [31,39,43,52]. The morphology of DCPD after immersion in SBF was observed to retain part of the pointed lamellar morphology with rough edges. Increasing the contact time with SGF causes changes in the crystalline order, such as the unit cell parameters, and an increase in crystallinity [17,30]. Figure 7d shows the surface of the DCPD after immersion in the SBF/SGF mixture. Changes are observed in the stacked plates, where the surface becomes irregular [41,45,48].

**Figure 8:**
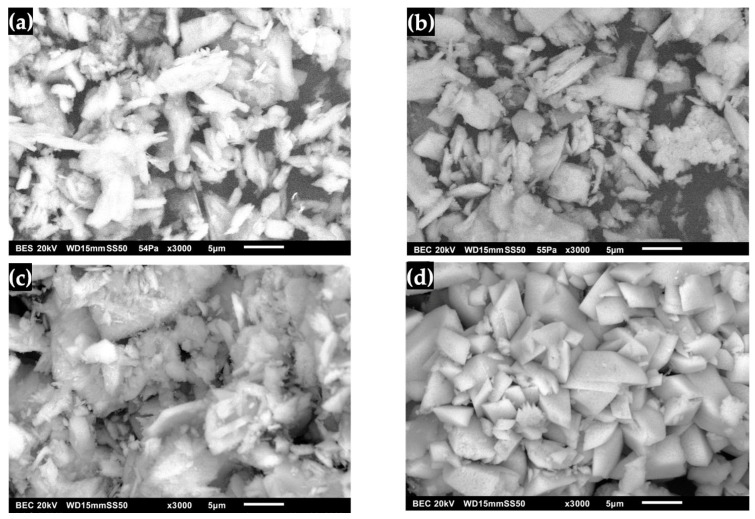
DCPD morphology with WL: (**a**) synthesized, (**b**) immersed in SBF, (**c**) immersed in SGF and (**d**) immersed in SBF/SGF.

The structure of synthesized DCPD with WL morphology is observed in Figure 8a, where agglomerated granulated powders can be distinguished that form the characteristic water lily structure of the surface [20,42,52,53]. The changes in the morphology of DCPD after its immersion in SBF are observed in Figure 8b, where it has the characteristic form of apatite, which begins to decompose, giving way to the formation of pointed plates with a rough surface [23,39,54,55]. In the case of immersion of DCPD in SGF (Figure 8c), an amorphous form with pointed crystals was observed that present a dispersion of DCPD crystals in the strong acid solution and the action of the enzyme present in the simulated fluid [23,56]. This indicates that the interaction of SGF with DCPD can result in changes on the surface of the biomaterial due to the formation of water-soluble salts, which lead to the formation of HA plates. Figure 8d shows DCPD after immersion in SBF/SGF, in which a granulated and agglomerated morphology is observed that is initially similar to a water lily and later transforms into an irregular rectangular shape [23,48]. This could be due to the change in the presence of Ca^2+^ ions due to the erosion of calcium phosphates, which is due to factors in the materials that regulate their dissolution under physiological conditions [23,25,27,28]. Given this difference in microstructure, the transformation with DMEM of DCPD to OCP can have a faster reaction rate on the FP surface than on the WL surface, as this form does not allow it to transform homogeneously into OCP [29].

### 2.5. Energy-Dispersive Spectroscopy (EDS) of Synthesized DCPD Before and After Immersion in Simulated Fluids

The elemental chemical composition of DCPD before and after immersion in physiological fluids is shown in Table 7, where the presence of C, O, P, Ca, and Na, among other elements, can be noted. The Ca/P ratio of the biomaterials had significant changes after their interaction with physiological fluids. In the case of DCPD-FP, a Ca/P ratio equal to 1.16 was observed, indicating the presence of amorphous calcium phosphates (ACPs) in the apatite. After immersion in SBF and SBF/SGF, the Ca/P ratio is 1.11 and 1.10, respectively. This indicates a change in the structure when it is transformed into calcium-deficient hydroxyapatite (CDHA) due to the presence of ions provided by SBF [2,3,56,57]. In the case of immersion in SGF, it is observed that the Ca/P ratio increases up to 1.26, which shows that the structure has stability with respect to this fluid [2,3,57]. It can be observed that the presence of C increases by around 29.74%, 73.85% and 15.23% after the interaction with SBF, SGF and SBF/SGF, respectively. On the other hand, the presence of O in the biomaterial after immersion in SGF remains practically the same as in DCPD. This indicates that it presents adequate stability in the presence of ions and pancreatin from the simulated fluid [2,3,57]. This is due to the precipitation of several CaP phases that depend on temperature and pH [57]. The Ca/P ratio of the biomaterials had significant changes after their interaction with physiological fluids. The Ca/P ratio for DCPD-WL has a value equal to 1.19, which indicates the presence of ACPs in the apatite. After immersion in SBF, the Ca/P ratio is 1.11; this indicates a change in the structure when it transforms into CDHA due to the presence of ions provided by SBF [2,3,30,57]. In the case of immersion in SGF and SBF/SGF, it is observed that the Ca/P ratio decreases to 1.06 and 1.08, respectively, indicating that the structure exhibits stability in these fluids [2,3,57]. In the case of the interaction between DCPD-WL and SBF and SBF/SGF, a decrease of approximately 35.88% in C and 29.62% in O was observed. These changes indicate different behaviors in the interaction of the biomaterial with the ions present in the different physiological fluids during the immersion process [2,3,30,57]. The different concentrations of ions present on the surface of each of the biomaterials can be directly related to the changes in the bands of the bonds of the functional groups observed in ATR-FTIR [2,3,30,57].

### 2.6. Scanning Electron Microscopy (SEM) of Synthesized OCP Before and After Immersion in Simulated Fluids

**Figure 9:**
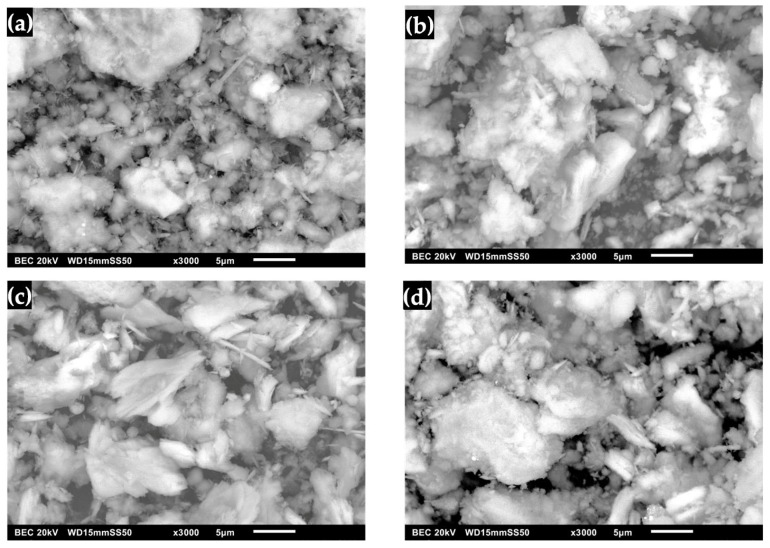
OCP (FP) micrographs: (**a**) synthesized, (**b**) immersed in SBF, (**c**) immersed in SGF and (**d**) immersed in SBF/SGF.

The transformation of DCPD to OCP with FP morphology is shown in Figure 9. It was observed that it no longer exhibits the shape of pointed lamellar particles, indicating that the surface morphology changed after immersion in DMEM (Figure 9a) [7,8,17,21,24,26]. Figure 9b shows the OCP after immersion in SBF, where the presence of a coating of lamellar crystals with round and irregular shapes that increased in size is observed [17,20]. This phenomenon is due to factors such as immersion time, temperature and pH, which caused crystal growth that contributed to the formation of a defined morphology aligned with the lamellar structure [20,24,26,27,29]. Furthermore, the use of an SBF solution leads to the formation of apatites as a bioactive coating [23,24,26]. In the micrograph of OCP after immersion in SGF (Figure 9c), the presence of plate-like shapes with sharp edges was observed, which allowed the formation of OCP crystals. This suggests that a progressive layer of crystallization and thickening occurred. This is associated with the enhanced formation of crystalline structures on the surface, with an increase in the amount of deposited material during prolonged immersion [20,23,24,27]. Upon immersing the obtained OCP in SBF/SGF, the formation of a network of plates and peaks was observed (Figure 9d). This can be attributed to the fact that the OCP can grow without transforming into HA. These results suggest that the driving force behind OCP formation depends on the concentration of CaCl_2_, HCO_3_ and other ions present in the solution, which play an important role in the kinetics of CaP phase formation [23,24,26,28,29,30].

**Figure 10:**
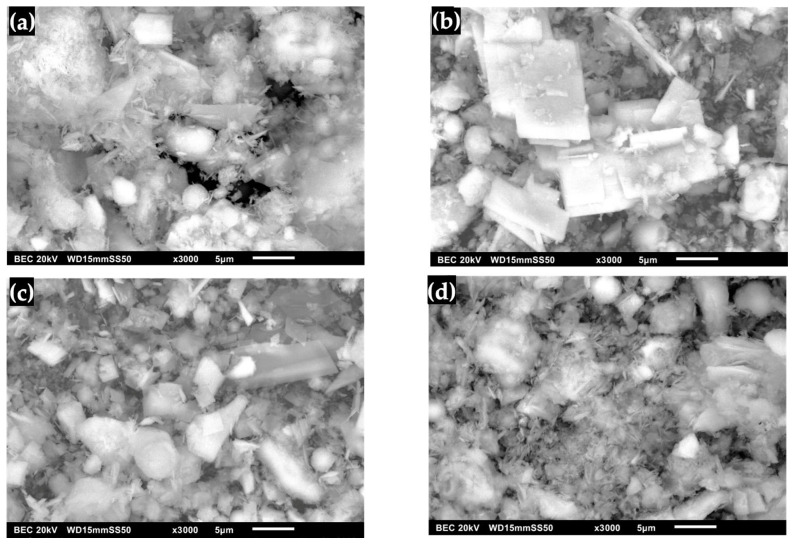
OCP (WL) micrographs: (**a**) synthesized, (**b**) immersed in SBF, (**c**) immersed in SGF and (**d**) immersed in SBF/SGF.

The transformation of OCP from DCPD with FP morphology is shown in Figure 10. The water lily shape characteristic of apatite is no longer observed. Instead, a smooth surface corresponding to the intrinsic shape of the OCP crystal can be seen, indicating the homogeneity of the biomaterial [7,8,23,26,40,50]. Figure 10b shows the OCP after immersion in SBF, where the formation of irregularly shaped lamellar structures that have increased in size is observed [23,25]. This process is influenced by immersion time, temperature and pH, allowing the growth of a defined morphology aligned with the laminar structure [23,25,31,40,50,54]. Figure 10c shows the micrograph of OCP after immersion with SGF, where pointed plates are present, allowing the formation of OCP crystals; this suggests that coating and coarsening occurred in OCP. This is associated with the formation of crystalline structures on the surface with an increase in the amount of deposited material during the immersion time [23,25,31,40]. Immersion of OCP in SBF/SGF is observed in Figure 10d, where a network of peaks is presented that can be attributed to the formation of HA [31,40,50,56]. This indicates that the concentration of HCO^3−^ and Ca^2+^ directly influences the formation of OCP [28,29,31,40,50,56].

### 2.7. Energy-Dispersive Spectroscopy (EDS) of Synthesized OCP Before and After Immersion in Simulated Fluids

The elemental chemical composition of DCPD before and after immersion in physiological fluids is shown in Table 8, where the elemental chemical composition of OCP-FP obtained has a Ca/P ratio equal to 1.31, which shows that the transformation of DCPD-FP was obtained and the Ca/P value of OCP is very close to the stoichiometric value of the apatite (1.33) [23,57]. OCP was immersed in SBF and SBF/SGF, where the Ca/P ratio was 1.30, indicating that it has good stability over time when immersed in this fluid. On the other hand, after immersion in SGF, the Ca/P ratio was 1.36 and 1.29. In addition, it was observed that the presence of C increases by 38.32% with interaction with SGF. Interaction with SBF and SBF/SGF resulted in decreases of 28.58% and 13.16%, respectively. The presence of O in the OCP after immersion in SGF remained essentially constant, indicating adequate stability in the presence of ions and pancreatin in the simulated fluid. The OCP immersed in SBF and SBF/SGF showed a decrease in the presence of O of 41.41% and an increase of 13.66%, respectively. For the LPA of OCP obtained, it is observed that the Ca/P value was 1.33; the transformation of DCPD into OCP and the Ca/P values after immersion in the simulated fluids are presented in Table 8 [17,51]. After immersion of OCP in SBF, SGF and SBF/SGF, Ca/P ratios of 1.31, 1.32 and 1.34 were obtained, indicating stability in the fluids throughout the immersion time [2,3,20,49]. It was observed that the presence of C in OCP-WL after interaction with SBF and SGF increased almost 2.5 times compared to that of the apatite before immersion. After immersion in SBF/SGF, a 1.5-fold increase was observed. It was observed that the O present in OCP after immersion in SBF and SGF decreased by 20.43% and 18.85%, respectively, compared to the apatite before immersion, and, in the case of immersion in SBF/SGF, the presence of O remained stable. The biological precursor to apatite formation in humans is OCP, which exhibits a water lily-like morphology [21,31,52]. However, the increased O and C values of OCP can be attributed to the loss of HPO_4_^−^. Values greater than 1.33 in the Ca/P ratio can be attributed to a defective structure in OCP crystals [2,3,7,8,50,53,57].

### 2.8. X-Ray Diffraction (XRD) of Synthesized DCPD Before and After Immersion in Simulated Fluids

**Figure 11:**
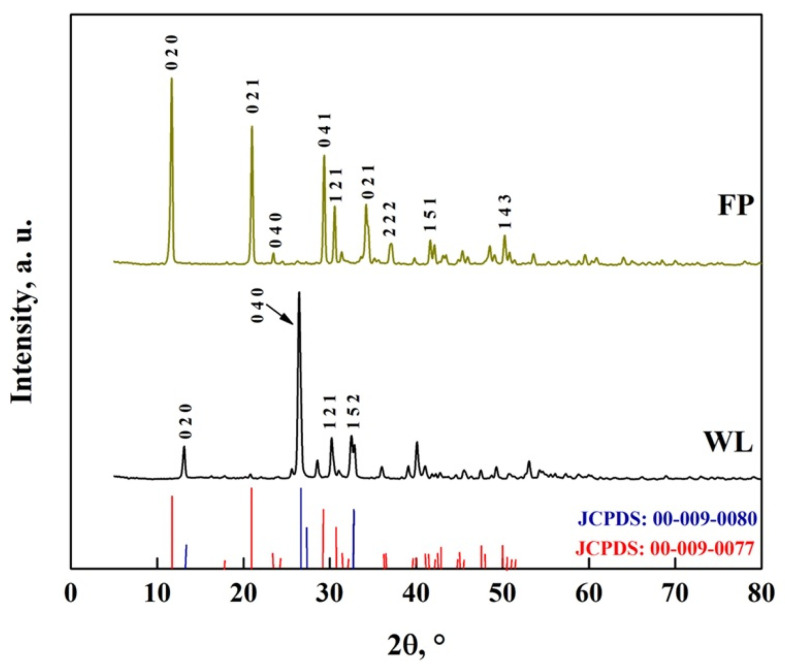
XRD pattern of DCPD-FP and DCPD-WL.

The crystal structure of DCPD-FP and DCPD-WL is shown in Figure 11. In the DCPD-FP pattern, peaks are observed at 11.8°, 21°, 23.2°, 29.4°, 30.5°, 31.6° and 34.4°, while in the DCPD-WL pattern, peaks are observed at 13.1°, 25.5°, 26.6°, 31.3°, 32.6° and 41°, with similar peaks observed in the patterns at 37.2°, 41.7° and 50.4° [10,26,52]. These peaks are characteristic of DCPD (JCPDS: 00-009-0077) and DCPA (JCPDS: 00-009-0080), which indicates that in both synthesized biomaterials, there is a mixture of apatites. It is also observed that the DCPD-FP peaks are more intense than those of DCPD-WL, which indicates that there is a different particle size and a greater presence of DCPD in the biomaterial [10,26,52,53,54,55,56,57,58,59,60,61,62,63,64].

**Figure 12:**
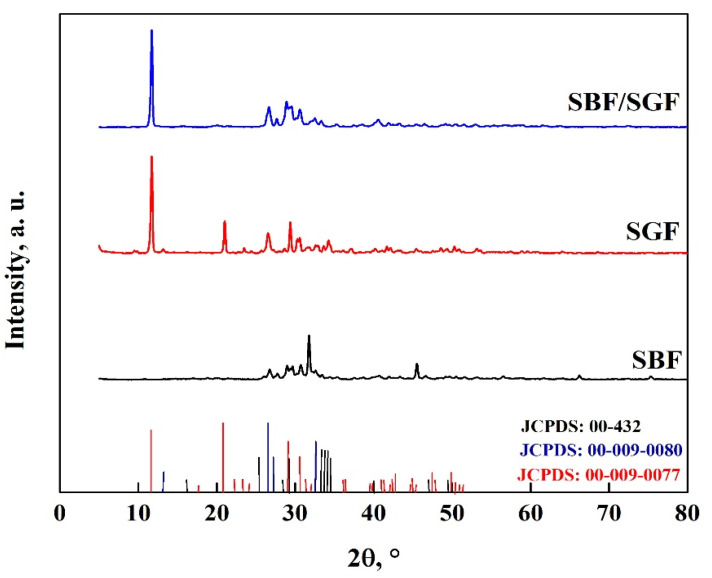
XRD pattern of DCPD-FP after immersion in simulated fluids.

Figure 12 shows the XRD pattern of DCPD-FP after immersion in simulated fluids, where peaks appear at 26.4°, 27.8°, 28.9°, 32.4° and 33.2°, corresponding to the presence of HA particles (JCDPS: 00-423) in the biomaterial [22,64,65,66]. Other peaks observed with the different fluids, associated with the presence of HA, include 26.2° and 45.6° for SBF, 13.4° for SGF, and 28.9° and 33.5° for the SBF/SGF mixture, indicating the transformation of DCPD into HA [22,59,65,66]. Additionally, the disappearance of peaks at 11.8°, 21°, 23.2°, 34.4° and 37.2° after interaction with the simulated fluids suggests a modification of the DCPD crystalline structure [22,59,64,65,66].

**Figure 13:**
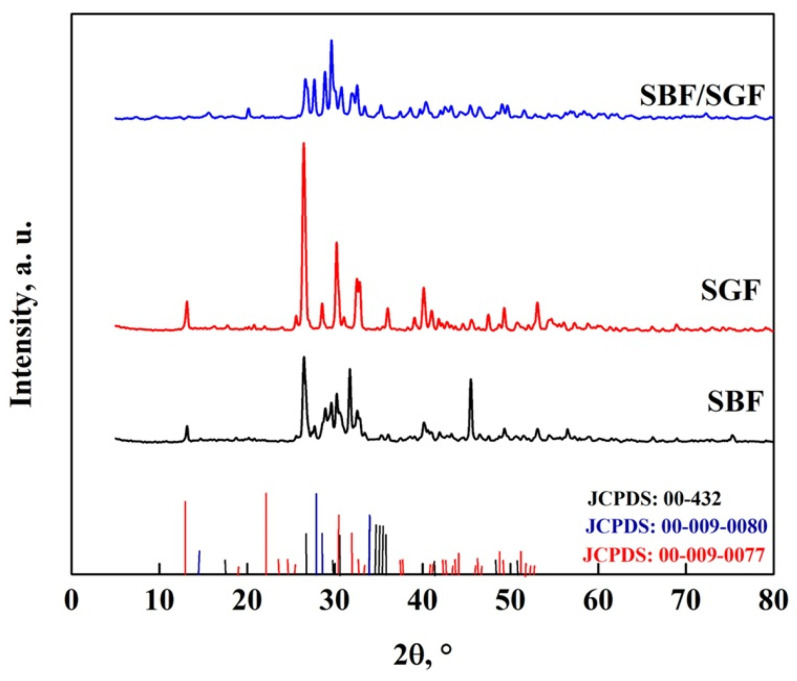
XRD pattern of DCPD-WL after immersion in simulated fluids.

The XRD pattern of DCPD-WL after immersion in the simulated fluids is shown in Figure 13, where peaks appear at 27.8°, 28.9°, 33.5°, 38.6 and 49.4°, corresponding to the production of HA particles [10,26,59,60,61,62,63]. In addition, peaks related to the presence of HA and the transition from DCPA to DCPD appear in the different simulated fluids: 18.8° and 31.9° in SBF; 17.6°, 41.8°, 44.6° and 50.7° in SGF; and 15.8°, 35.2°, 37.2°, 38.7°, 43.2° and 46.4° in SBF/SGF [10,26,59,60,61,62,63]. With the interaction with SBF and SBF/SGF, the disappearance of peaks in the DCPD pattern was observed. In SBF, the peaks were 20.9° and 41°, and for SBF/SGF, they were 11.8°, 21°, 23.2°, 34.4° and 37.2°. During immersion in SGF, no significant changes were observed in the DCPD pattern, which indicates that there is greater crystalline stability than in the interaction with the other two fluids [10,26,59].

### 2.9. X-Ray Diffraction (XRD) of Synthesized OCP Before and After Immersion in Simulated Fluids

**Figure 14:**
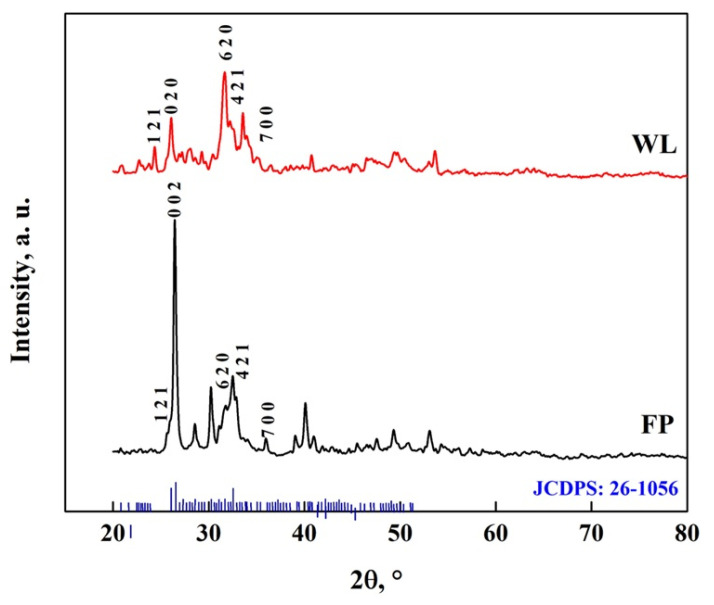
XRD pattern of OCP with FP and WL morphology.

Through Rietveld analysis, a transformation fraction of OCP-WL of 61.2% for WL and 81.2% for FP was obtained, which indicates that a simpler morphology promotes a faster transformation rate [67,68]. The transformation of DCPD to OCP due to the interaction with DMEM is shown in Figure 14, where characteristic peaks of OCP (JCDPS: 26-1056) can be observed at 30.2°, 31.6°, 32.4°, 38.9°, 40.2°, 49.5° and 53.2° [7,8,50,58,59,67,68]. The peak observed at 26.5° is attributed to CaCO_3_ particles; as it is in OCP-FP form, this is the one with the highest intensity [59,60]. In addition, several peaks appear at different positions of DCPD; in the case of OCP-FP, peaks are observed at 25.7°, 28.5°, 35.9°, 41.1°, 47.5° and 51°, while for OCP-WL, peaks are observed at 21.1°, 22.8°, 24.4°, 27.9°, 29.2°, 33.5°, 35.2°, 45.4°, 46.9° and 50.4°. These peaks correspond to the crystal structure of OCP [10,21,31,50,51,52,53,61,62]. This result indicates that DCPD transforms into OCP between 48 and 168 h [57,58,67,68].

**Figure 15:**
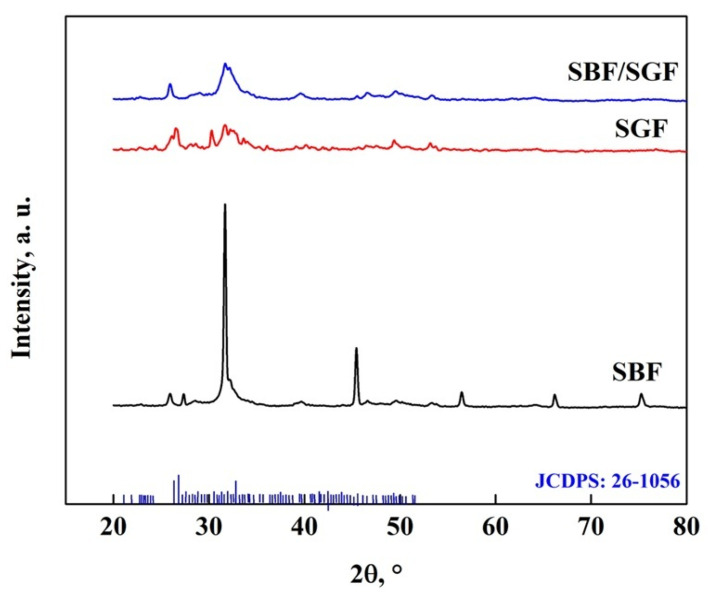
XRD pattern of OCP-FP after immersion in simulated fluids.

The interaction between the OCP-FP and the simulated fluids is shown in Figure 15, where it is observed that the simulated fluids cause changes in the crystallography of the apatite. Peaks appear at 27.7°, 29.6° and 45.6°, which are due to the HA particles obtained with the simulated fluids [7,8,25,40,47,69]. In addition, peaks related to DCPD particles were observed at 42°, 46.5° and 56.5° in the SBF immersion, and with the other fluids, the particles were not formed [7,8,25,45,56,57,58,67,68,69]. A peak at 51° also disappears, indicating that there are changes in the crystal structure of the OCP [7,25,40,53,57,58,68,69]. The stability of the biomaterial is related to the modification of the pattern in the different fluids, which has a greater impact on stability in SBF > SBF/SGF > SGF [7,25,40,57,58,59,61,68,69,70].

**Figure 16:**
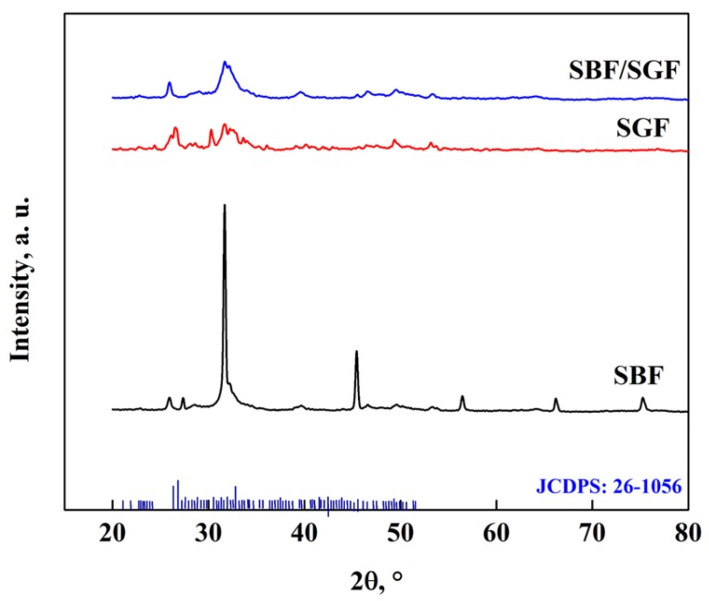
XRD pattern of OCP (WL) after immersion in simulated fluids.

Figure 16 shows the XRD pattern of the OCP-WL after immersion in simulated fluids, where the appearance of peaks at 25.9° and 39.6° related to HA and DCPD particles, respectively, can be observed [7,40,50,58,59,68,69]. In addition, after immersion in SGF, a peak at 22° appears, which corresponds to the presence of DCPD particles [7,40,50,53,58,68,69]. The changes that occur in the OCP pattern in the different fluids show the disappearance of the peaks at 21.1°, 22.8°, 24.4°, 27.9°, 33.5°, 35.2°, 38.5°, 40.7° and 53°, which indicates changes in the crystalline structure of the OCP [7,25,40,57,70]. The stability of the OCP with the different simulated fluids follows the following behavior of greater impact: SBF/SGF > SBF > SGF [7,25,40,57,70].

## 3. Methods and Materials

### 3.1. DCPD Synthesis in Water Lily-Shaped Crystals (WL)

DCPD with WL morphology was synthesized by dissolving 40 g of ammonium dihydrogen phosphate (NH_4_H_2_PO_4_, Jalmek 100%) in 340 mL of distilled water. The solution was stirred by adding 10 g of calcium carbonate (CaCO_3_, Jalmek 100%). The suspension formed was stirred at 500 rpm at room temperature for 30 min. After this time had elapsed, the suspension particles were recovered and washed with 750 mL of distilled water. Finally, the powders obtained were dried for 24 h at 75 °C in a static air oven [6,22,40]. DCPD in its WL form was stored in a desiccator until use, and the synthesis was performed in quadruplicate.

### 3.2. DCPD Synthesis in Flat Plate-Shaped Crystals (FP)

For the formation of DCPD crystals in FP form, two solutions were prepared. In solution A, 0.835 g of monopotassium phosphate (KH_2_PO_4_) and 3.013 g of sodium hydrogen phosphate (Na_2_HPO_4_) were dissolved in 700 mL of distilled water. In solution B, 4.014 g of calcium chloride dihydrate (CaCl_2_·2H_2_O) was dissolved in 200 mL of distilled water. Then, solution B was rapidly added to solution A under stirring at 500 rpm at room temperature for 80 min. The solids recovered by filtration were dried for 24 h at 65 °C [22,26,44]. DCPD in its FP form was stored in a desiccator until use, and the synthesis was performed in quadruplicate.

### 3.3. Transformation of DCPD in WL and FP Shape into OCP Using DMEM Solution

For the transformation of DCPD in WL form to OCP, 1 g of DCPD was placed in a beaker with 50 mL of DMEM solution at a temperature of 36.5 °C for 24, 48 and 164 h to determine the effect of the contact time of DMEM solution on DCPD. The solutions were then filtered, washed with 500 mL of deionized water and left to dry at 65 °C for 24 h. For the transformation of DCPD in FP form to OCP in DMEM solution, 1 g of DCPD was placed in a beaker with 50 mL of DMEM solution at 36.5 °C for 24, 48 and 164 h to determine the effect of contact time of DMEM solution on DCPD. The solutions were then filtered, washed with 500 mL of deionized water and allowed to dry at 65 °C for 24 h. They were subsequently stored until use [9,22,26,44,50]. DCPD transformed into OCP was stored in a desiccator until use, and the synthesis was performed in triplicate.

### 3.4. Preparation of Body Fluid (SBF) and Simulated Gastric Fluid (SGF)

Human or simulated body extracellular fluid (SBF) solutions were prepared. This solution contains ions such as Cl^−^, HPO_4_^−^, HCO_3_^2−^, SO_2_^4−^, Na^+^, K^+^, Mg^2+^ and Ca^2+^ dissolved in deionized water with a Ca/P ratio of 2, as shown in Table 9 [11,61]. Also, a HCl solution (0.1 M) was prepared to maintain a pH of 7.4 [26,48,70].

For the interaction of DCPD with SGF solution, concentrated NaCl, pepsin and HCl were dissolved in deionized water in the amounts shown in Table 10, maintaining the pH of the solution at 1.2 [54,56].

### 3.5. Study of DCPD and OCP Changes After Immersion in SBF, SGF and SBF/SGF

Each fluid solution was subjected to an autoclave sterilization process at 121 °C and 1 atm pressure for 15 min. After sterilization, a 1/10 ratio (weight/volume) of OCP or DCPD with SGF, SBF and SBF/SGF, respectively, was placed under vigorous stirring at 37 °C for 12 h. After this time, the powder was filtered and dried for 24 h at 75 °C in a static air oven and subsequently weighed [33,44,56,72]. The immersion process was repeated in triplicate for each simulated fluid and for each type of apatite.

### 3.6. Characterization of DCPD and OCP

To perform DCPD and OCP analysis before and after immersion in physiological fluids, attenuated total reflectance Fourier transform infrared spectroscopy (ATR-FTIR) was used in the wavenumber range of 4000 to 400 cm^−1^ with a Thermo Scientific Nicolet iS10 analyzer (Waltham, MA, USA). A total of 32 scans were obtained with a resolution of 4 cm^−1^. In addition, X-ray diffraction (XRD) patterns were obtained with a diffractometer (Ultima IV Rigaku, Cedar Park, TX, USA), with a radiation (Cu Kα) of 1.5406 Å measuring from 4° to 80° in 2θ with a step size of 0.03°. Scanning electron microscopy (SEM) images and energy-dispersive X-ray spectroscopy (EDS-EDX) values were obtained using a JOEL spectrometer (6510 pus, Peabody, MA, USA) following the ASTM E 1508 standard [73].

## 4. Conclusions

In this study, DCPD-FP and DCPD-WL were synthesized, from which the functional groups, morphology and characteristic crystalline structure of the apatite were identified, indicating that the synthesized DCPD has properties comparable to those of commercial biomaterials. Stability was studied when immersed in simulated fluids, where it was observed that DCPD-FP presents less degradation during the immersion time than DCPD-WL. In addition, modifications in the crystalline structure were observed after immersion in the simulated fluids mainly due to the formation of HA particles and changes in the morphology of the DCPD. The percentage of transformation of DCPD to OCP depended on the morphology. In addition, the formation rate depended on the morphology, as DCPD-FP was faster in transformation than DCPD-WL because the structure allows a more efficient interaction with DMEM. The functional groups, morphology and the crystalline structure of the OCP obtained were observed. Upon immersion in SBF, the formation of HA particles was observed, and apatite degradation was more significant (>70%) in SGF, suggesting that the biomaterial may be a good candidate as an excipient for drug delivery in living organisms.

## Figures and Tables

**Table 1:** Assignment of the ATR-FTIR bands of both DCPD structures.

WL	FP	Description	Reference
cm^−1^			
3543	3541	O-H extension in the H_2_O molecule	[14,33,34,35]
3480	3470		
3279	3254		
3153	3152		
2979		O-H stretching	
	2926		
2876	2870		
1720 ^w^	1717 ^w^	Vibration of the HPO_4_^2−^ groups	[35,36,37]
	1644	O-H bending and rotation mode in the H_2_O molecule	[33,36,41]
1625			
1215		Stretching due to P-O	[20,35,37]
	1202		
1123	1122	Stretching vibrations ν′_6_ and ν″_6_ of the HPO_4_^−2^ groups	[35,36,41]
1062		Stretching of group PO_4_^3−^ and HPO_4_^2−^	[35,36,38,39]
	1050		
980	978	Asymmetric stretching of P-O	[20,33,34,37,38]
877		Asymmetric stretching of P-O-P	
785		Asymmetric stretching of P-O	
	773		
	651	Bending mode O-P-O	
622			
580	577	Vibrations of phosphoric acid bonds (H-O-) P=O	
530	527		

w = weak.

**Table 2:** Position and assignment of the ATR-FTIR bands of DCPD-WL after its interaction with simulated fluids.

BF	SGF	SBF/SBG	Description	Reference
cm^−1^				
	3527		O-H extension in the H_2_O molecule	[21,35,36,41,42,43,44]
3548	3476			
3282	3281			
	3149			
2969	2894		O-H stretching	
2866	2881			
1648	1652	1649	O-H bending and rotation mode in the H_2_O molecule	
1525		1527	O-H-O bending mode of H_2_O residual	
	1518			
		1214	P=O stretching of the PO_2_^3−^ groups	[35,36,37,38,43,44]
1207				
	1190		Bending mode (O-H) in the HPO_4_^2−^ groups	[35,36,37,38,41,43,44]
1185			P-OH stretching	[38]
		1163		
1146		1144	P=O stretching of the PO_2_^−^ groups	[20,35,36,37,38,41,42,44]
	1119		Stretching vibrations ν’_6_ and ν’’_6_ of the HPO_4_^2−^ groups	
1075			Stretching of the PO_4_^3−^ and HPO_4_^2−^ groups	[35,36,38,39]
		1063	P=O stretching from the PO_4_^3−^ bond	[35,37,38,43,44]
	1057		ν_3_ (P-O) stretching of the PO_4_^3−^ groups	[20,34,35,36,37,38,41,42,43]
1023		1023	ν_1_ stretching of the PO_4_^3−^ group	
981	985	981	Asymmetric stretching of P-O	
972			Asymmetric stretching of P-O-P	
		961		
		921	P=O stretching of the PO_4_^3−^ group	
877	876		Asymmetric stretching of P-O-P	
	862		P-O stretching in the PO_4_^3−^ group	[35,36,37,38,41,42,43,44]
	790		Release of O-H out of the bending plane	
715		719	P=O stretching of the PO_2_^−^ groups	
669	671	669	P-O deformation mode	[20,37,44]
612		612 ^w^	Acid phosphate bond (H-O-) P=O	
	606			
	575		P=O bending mode	
552		562		
	534		O-P-O(H) bending mode	[34,35,36,41,43,44]

w = weak.

**Table 3:** Position and assignment of ATR-FTIR bands of DCPD-FP after its interaction with simulated fluids.

SBF	SGF	SBF/SGF	Description	Reference
cm^−1^				
3625	3439		O-H stretching in the H_2_O molecule	[20,34,35,42,43,44]
3372	3390			
2960	2955		O-H stretching	
2865	2875			
1669			Bending and rotation mode of O-H in the H_2_O molecule	
	1639	1634		
	1575			
1515				
		1402	P-O-H bending mode	[35,36,37,38,41,43,44]
	1341	1341 ^w^		
	1292			
1207		1210	P=O stretching of the PO_2_^−^ groups	
1167			P-OH stretching	[38]
		1159	Stretching vibrations ν′_6_ and ν″_6_ of the HPO_4_^2−^ groups	[35,37,42,43,44]
1146			P=O stretching of the PO_2_^−^ groups	
		1139	Stretching vibrations ν′_6_ and ν″_6_ of the HPO_4_^2−^ groups	
	1126			
1074			O-H-O bending mode from the H_2_O residual	[20,35,37,44]
	1065	1068		
1023			Stretching of the PO_4_^3−^ (ν_1_) group	
	993		Asymmetric stretching of P-O	
981				
		940	P-O stretching in the PO_4_^3−^ group	[20,35,36,37,41,42,43,44]
931		928	Asymmetric stretching of P-O-P	
	890		P-O stretching in the PO_4_^3−^ group	
877			Asymmetric stretching of P-O-P	
715			P=O stretching of the PO_2_^−^ groups	
669	665		P-O deformation mode	
612 ^w^		613	Acid phosphate bond (H-O-) P=O	
	583		O-P-O(H) bending mode	[35,36,41,42,44]
	562			
553	534		P=O bending mode	[20,37,41]

w = weak.

**Table 4:** ATR-FTIR Band Assignment for OCP-WL and OCP-FP.

WL	FP	Description	Reference
cm^−1^			
3256	3253	Stretching of the O-H extension in the H_2_O molecule	[20,34,35,44]
1647	1640	Bending and rotation mode of O-H in the H_2_O molecule	[8,23,36,37,38,42]
	1558	Bending mode of CO_3_^2−^ (ν_3_)	
	1403	CO_3_^2−^ (ν_3_) stretching	
	1332	PO_4_^3−^ (ν_3_) stretching	
1293		Stretching of the P-O groups	
1200 ^w^			
	1167 ^w^	Bending mode of P-O	
1070 ^w^		P-O stretching	[23,35,37]
	1035	PO_4_^3−^ (ν_3_) stretching	
1020			
	963	ν_1_ (PO_4_^3−^) stretching	[4,7,8,23]
950		PO_4_^3−^ (ν_3_) stretching	[7,14,23,26,35,36,37,38,41]
909			
	881	Bending mode of CO_3_^2−^ (ν_3_)	
859		P-OH stretching	
	665 ^w^	Bending mode of PO_4_^3−^ (ν_4_)	[8,23,44]
606		Bending mode of (ν_4_) PO_4_^3−^	[23,35,43]
	595 ^w^	P-O(H) stretching	[4,8,23,43,44]
	584		
555		Bending mode of O-P-O(H)	[8,23,44]

w = weak.

**Table 5:** Position and assignment of ATR-FTIR bands of OCP-WL after its interaction with simulated fluids.

SBF	SGF	SBF/SBG	Description	Reference
cm^−1^				
		3340	O-H stretching in the H_2_O molecule	[35,37,43,47,48,49]
	3213			
1640		1644	Bending and rotation of O-H in the H_2_O molecule	[20,36,41,42,44]
	1635			
1550 ^w^	1553 ^w^		Bending mode of CO_3_^2−^ (ν_3_)	[35,41,48,49]
1465 ^w^		1470 ^w^	Stretching of CO_3_^2−^ (ν_3_)	
	1399	1398 ^w^	Bending mode in the plane P-O-H	[41,43]
	1327 ^s^			
		1214	P=O stretching of the groups PO_2_^−^	[36,37,38,41,43,44]
	1123 ^s^		Stretching of HPO_4_^2−^	[35,37,41,49]
1096 ^s^			Stretching of PO_4_^3−^ (ν_3_)	[35,43,44,49,50]
		1080		
1025	1020	1019		
953 ^w^	958 ^w^		Asymmetric stretching of P-O-P	[35,37]
		937		
881			Bending mode of CO_3_^2−^ (ν_3_)	[23,26,35,43,44]
877				
	856		Stretching of HPO_4_^2−^	
727		722	Bending of O-H out of the plane	[35]
		608	Acid phosphate bond (H-O-) P=O	[20,37,44]
604			Bending mode of PO_4_^3−^ (ν_3_)	[7,35,44,49]
	589		Bending mode of PO_4_^3−^ (ν_4_)	[23,35,41,44]
562				[20,37,44]
	558	558	Vibrations of O-P-O	[23,35,41,44]

w = weak; s = strong.

**Table 6:** Assignment of ATR-FTIR bands of OCP (FP) after its interaction with simulated fluids.

SBF	SGF	SBF/SBG	Description	Reference
cm^−1^				
		3322	O-H stretching in the H_2_O molecule	[4,34,35,44]
3298	3287			
	1640	1642	Bending and rotation mode of O-H in the H_2_O molecule	[14,36,41,42,43,44]
1655				
	1565 ^w^		Bending mode of CO_3_^2−^ (ν_3_)	[23,35,43]
1555 ^w^		1559		
	1450 ^w^	1457	Stretching of CO_3_^2−^ (ν_3_)	
		1405	Bending mode in the plane P-O-H	
1215 ^w^		1214	Stretching vibrations P=O of the PO_2_^−^ groups	[23,26,35,43,44,50]
		1119 ^w^	Stretching of PO_4_^3−^ (ν_3_)	
1153			Bending mode of P-OH	
1132 ^w^			Stretching of HPO_4_^2−^	
1081			Stretching of PO_4_^3−^ (ν_3_)	
	1086			[35,36,38,39]
	1015 ^w^			
1030		1026		[23,26,35,43,44,50]
	964 ^w^		Asymmetric stretching of P-O-P	[35,37]
938			Bending mode of CO_3_^2−^ (ν_2_)	[23,35,43]
881 ^w^	883 ^w^	883 ^w^		
877			Asymmetric stretching P-O-P	[20,34,35,37,38]
722			P=O stretching of the PO_2_^−^ groups	[35,36,37,38,43,44]
	669		Bending mode of PO_4_^3−^ (ν_4_)	[19,23,35,43,44,50]
600	598	595		
558		556	Bending mode of P=O	[20,37,44]

w = weak.

**Table 7:** Elemental analysis of DCPD with FP and WL morphology.

Biomaterials	C	O	Cl	Al	Na	Ca/P
**FP**	19.41	43.76	0.91	1.26	0	1.16 ± 0.08
SBF	14.535	43.24	0.43	1.4	1.4	1.10 ± 0.06
SGF	7.805	45.05	1.34	0	0	1.26 ± 0.07
SBF/SGF	49.02	44.17	1.09	0	0	1.11 ± 0.05
**WL**	7.25	55.57	0	0.78	0	1.19 ± 0.08
SBF	9.82	34.54	3.59	0	1.85	1.11 ± 0.06
SGF	14.08	52.68	0	0.4	1.44	1.06 ± 0.03
SBF/SGF	15.01	37.98	0	0.66	0	1.08 ± 0.04

**Table 8:** Elemental analysis of OCP obtained from DCPD with FP and WL morphology.

Biomaterials	C	O	Cl	Na	Mg	Ca/P
**OCP-FP**	17.25	43.3	0.31	0.52	0.23	1.31 ± 0.02
SBF	12.32	25.37	7.67	6.12	0.32	1.30 ± 0.01
SGF	23.86	44.71	0.67	0.35	0	1.36 ± 0.05
SBF/SGF	14.98	37.52	0.32	0.33	0	1.29 ± 0.03
**OCP-WL**	8.04	43.52	0	0.59	0	1.33 ± 0.04
SBF	19.11	34.63	4.81	4.58	0.21	1.31 ± 0.03
SGF	21.05	35.32	0.41	0.23	0	1.32 ± 0.05
SBF/SGF	11.87	43.46	1.3	2.04	0.23	1.34 ± 0.06

**Table 9:** Preparation of 1 L of SBF solution [71].

Compound	SBF (10^−3^ mol/L)
NaCl	142.9
NaHCO_3_	4.3
KCl	7.4
K_2_HPO_4_	1.3
MgCl_2_	3.3
CaCl_2_	2.6
Na_2_SO_4_	0.51
Tris	5.05

**Table 10:** Preparation of 1 L of SGF and SIF solution.

Compound	SIF, (10^−3^ mol/L)
NaOH	38.2
Pancreatin	2 *
KH_2_PO_4_	0.050
HCl	226.6

* gram.

## Data Availability

The original contributions presented in this study are included in the article/Supplementary Material. Further inquiries can be directed to the corresponding author.

## Supplementary Materials

The following supporting information can be downloaded at: https://www.mdpi.com/article/10.3390/molecules30173631/s1, Figure S1. ATR-FTIR spectra of the transformation of DCPD-FP to OCP-FP. Figure S2. ATR-FTIR spectra of the transformation of DCPD-WL to OCP-WL.

## References

[1] Anaya-Barajas D., Aguilar-Pliego J., González-Vélez V., Vélez-Tirado M. (2019). Biomaterials for Bone Tissue Regeneration Extracted from Fish Wastes. Rev. Mex. Ing. Biomed..

[2] Dorozhkin S.V. (2016). Calcium orthophosphates (CaPO_4_): Occurrence and properties. Prog. Biomater..

[3] Canilla M., Pena P., H de Aza A., Rodriguex M.A. (2017). Calcium phosphates for biomedical applications. Céramica Y Vidr..

[4] Cheng L., Lin T., Khalaf A.T., Zhang Y., He H., Yang L., Yan S., Zhu J., Shi Z. (2021). The preparation and application of calcium phosphate biomedical composites in filling of weight-bearing bone defects. Sci. Rep..

[5] Hurle K., Oliveira J.M., Reis R.L., Pina S., Goetz-Neunhoeffer F. (2021). Ion-doped Brushite Cements for Bone Regeneration. Acta Biomater..

[6] Barua R., Daly-Seiler C.S., Chenreghanianzabi Y., Markel D., Li Y., Zhou M., Ren W. (2021). Comparing the physicochemical properties of dicalcium phosphate dihydrate (DCPD) and polymeric DCPD (P-DCPD) cement particles. J. Biomed. Mater. Res. Part B Appl. Biomater..

[7] Teterina A.Y., Smirnov I.V., Fadeeva I.S., Fadeev R.S., Smirnova P.V., Minaychv V.V., Kobyakova M.I., Fedotov A.Y., Barinov S.M., Komlv V.S. (2021). Octacalcium Phosphate for Bon Tissue Engineering Synthesis Modification and In Vitro Biocompatibility Assessment. Int. J. Mol. Sci..

[8] Shen D., Horiuchi N., Nozaki S., Miyashin M., Yamashita K., Nagai A. (2017). Synthesis and enhanced bone regeneration of carbonate substituted octacalcium phosphate. Bio-Medical Mater. Eng..

[9] Viswanath B., Ravishankar N. (2008). Controlled synthesis of plate-shaped hydroxyapatite and implications for the morphology of the apatite phase in bone. Biomaterials.

[10] Kim Y., Lee S.Y., Roh Y., Lee J., Kim J., Lee Y., Bang J., Lee Y.J. (2015). Optimizing calcium phosphates by the control of pH and temperature via wet precipitation. J. Nanosci. Nanotechnol..

[11] Habraken W.J., Wolke J.G., Jansen J.A. (2007). Ceramic composites as matrices and scaffolds for drug delivery in tissue engineering. Adv. Drug Deliv. Rev..

[12] Alshaaer M., Kailani M.H., Ababneh N., Abu Mallouh S.A., Sweileh B., Awidi A. (2017). Fabrication of porous bioceramics for bone tissue applicationsusing luffa cylindrical fibres (LCF) as template. Process. Appl. Ceram..

[13] Dong D., Su H., Li X., Jiang H., Shen Z., Liu Y., Yu M., Guo Y., Yang P., Zhang Z. (2024). Influences of curing depth and layer thickness on forming accuracy and mechanical properties of biphasic calcium phosphate bioceramics fabricated by vat photopolymerization. J. Manuf. Process..

[14] Somers N., Jean F., Lasgorceix M., Preux N., Delmotte C., Boilet L., Petit F., Leriche A. (2023). Fabrication of doped β-tricalcium phosphate bioceramics by Direct Ink Writing for bone repair applications. J. Eur. Ceram. Soc..

[15] Safitri N., Rauf N., Tahir D. (2023). Enhancing drug loading and release with hydroxyapatite nanoparticles for efficient drug delivery: A review synthesis methods, surface ion effects, and clinical prospects. J. Drug Deliv. Sci. Technol..

[16] Mashak A., Bazraee S., Mobedi H. (2022). Advances in drug delivery and biomedical applications of hydroxyapatite-based systems: A review. Bull. Mater. Sci..

[17] Kolmas J., Krukowski S., Laskus A., Jurkitewicz M. (2016). Synthetic hydroxyapatite in pharmaceutical applications. Ceram. Int..

[18] Flores Valdez J.D., Sáenz Galindo A., López Badillo C.M., Castañeda Facio A.O., Acuña Vazquez P. (2022). Hydroxyapatite and Biopolymer Composites with Promising Biomedical Applications. Rev. Mex. De Ing. Biomédica.

[19] Khalifehzadeh R., Arami H. (2020). Biodegradable calcium phosphate nanoparticles for cancer therapy. Adv. Colloid Interface Sci..

[20] Boanini E., Silingardi F., Gazzano M., Bigi A. (2021). Synthesis and Hydrolysis of Brushite (DCPD): The Role of Ionic Substitution. Cryst. Growth Des..

[21] Zhao Z., Zhao J., Peng C., Duan X., Deng M., Wen J. (2023). Comparative analysis of petal epidermal wax composition and loss-water resistance in five cut lily cultivars (Lilium spp.). Sci. Hortic..

[22] Mandel S., Tas A.C. (2010). Brushite (CaHPO_4_2H_2_O) to octacalcium phosphate (Ca(HPO_4_)_2_(PO_4_)_4_5H_2_O) transformation in DMEM solutions. Mater. Sci. Eng. C.

[23] Rabadjieva D., Sezanova K., Gergulova R., Titorenkova R., Tepavitcharova S. (2020). Precipoitation and pase transformation of dicalcium phosphate dehydrate in electrolyte solutions of simulated body fluids: Thermodynamic modeling and kinetic studies. J. Biomed. Mater. Res. Part A.

[24] Shiwaku Y., Suzuki O. (2020). Octacalcium phosphate effects on the systemic and local factors that regulate bone-cell activity. Octacalcium Phosphate Biomater..

[25] Ban S., Jinde T., Hasegawa J. (1992). Phase Transformation of Octacalcium Phosphate in vivo and in vitro. Dent. Mater. J..

[26] Komlev V.S., Bozo I.I., Deev R.V., Gurin A.N. (2020). Bioactivity and effect of bone formation for octacalcium phosphate ceramics. Octacalcium Phosphate Biomater..

[27] Carino A., Ludwig C., Cervellino A., Müller E., Testino A. (2018). Formation and transformation of calcium phosphate phases under biologically relevant conditions: Experiments and modelling. Acta Biomater..

[28] Perez L., Shyu L.J., Nancollas G.H. (1989). The Phase Transformation of Calcium Phosphate Dehydrate into Octacalcium Phosphate in Aqueous Suspensions. Colloids Surf..

[29] Temizel N., Girisken G., Tas A.C. (2011). Accelerated transformation of brushite to octacalcium phosphate in new biomineralization media between 36.5 °C and 80 °C. Mater. Sci. Eng. C.

[30] Oliveira C., Georgieva P., Rocha F., Ferreira A., de Azevedo S.F. (2007). Dynamical model of brushite precipitation. J. Cryst. Growth.

[31] Lu X., Leng Y. (2005). Theoretical analysis of calcium phosphate precipitation in simulated body fluid. Biomaterials.

[32] Suzuki O. (2013). Octacalcium phosphate (OCP)-based bone substitute materials. Jpn. Dent. Sci. Rev..

[33] Jalota S., Bhaduri S.B., Tas A.C. (2008). Using a synthetic body fluid (SBF) solution of 27 mM HCO_3_^−^ to make bone substitutes more osteointegrative. Mater. Sci. Eng. C Mater. Biol. Appl..

[34] Kumar A.R., Kalainathan S. (2010). Microhardness studies on calcium hydrogen phosphate (brushite) crystals. Mater. Res. Bull..

[35] Arifuzzaman S.M., Rohani S. (2004). Experimental study of brushite precipitation. J. Cryst. Growth.

[36] Abs El-Ghany M.F., El-Kherbawy M.I., Abdel-Aal Y.A., El-Dek S.I., El-Baky T.A. (2021). Comparative Study between Traditional and Nano Calcium Phosphate Fertilizaers on Growth ann Prodiuction of Snap Bean (*Phaseolus vulgaris* L.) Plant. Nanomaterials.

[37] Suryawanshi V.B., Chaudhari R.T. (2014). Growth and characterization of Agar Gel Grown Brushite Crystals. Indian J. Mater. Sci..

[38] Dabiri S.M.H., Lagazzo A., Barberis F., Shayganpour A., Finocchio E., Pastorino L. (2017). New in-situ synthetized hydrogel composite based on alginate and brushite as a potential pH sensitive drug delivery system. Carbohydr. Polym..

[39] Touny A.H., Dawkins H., Zhou H., Bhaduri S.B. (2011). Hydrolysis of monetite/chitosan composites in α-MEM and SBF solutions. J. Mater. Sci. Mater. Med..

[40] Ito N., Kamitakahara M., Yoshimura M., Ioku K. (2014). Importance of nucleation in transformation of octacalcium phosphate to hydroxyapatite. Mater. Sci. Eng. C.

[41] Monsour S.F., El-dek S.I., Ahmed M.A., Abd-Elwahab S.M., Ahmed M.K. (2016). Effect of preparation conditions on the nanostructure of hydroxyapatite and brushite phases. Appl. Nanosci..

[42] Aquib M., Alomar M., Answer A., Naseem K., Javaid A., Intisar A., Khan S., Ajaz H., Khan I.H. (2024). Synthesis characterization and antimicrobial analysis of metal-doped (Zn^2+^ and Ag^+^) brushite powder for bone regeneration. Mater. Chem. Phys..

[43] Djošić M., Mišković-Stanković V., Kačarević-Popović Z., Jokić B., Bibić N., Mitrić M., Milonjić S., Jančić-Heinemann R., Stojanović J. (2009). Electichemical synthesis of nanosized monetite powder and its electrophoretic deposition on titanium. Colloids Surf. A Physicochem. Eng. Asp..

[44] Petrakova N.V., Teterina A.Y., Mikheeva P.V., Akhmedova S.A., Kuvshinova E.A., Sviridova I.K., Sergeeva N.S., Smirnov I.V., Fedotov A.Y., Kargin Y.F. (2021). In vitro study of octacalcium phosphate behavior in different model solutions. ACS Omega.

[45] Lim H.N., Kassim A., Huang N.M., Lee K.H., Syahida A., Chia C.H. (2010). High internal phase emulsion as reaction medium for precipitating brushite crystal. Ceram. Int..

[46] Kong J., Yu S. (2007). Fourier transform infrared spectroscopic analysis of protein secondary structures. Acta Biochim. Biophys. Sin..

[47] Disanto G., Benkert P., Lorscheider J., Mueller S., Vehoff J., Zecca C., Ramseier S., Achtnichts L., Findling O., Nedeltchev K. (2016). The Swiss Multiple Sclerosis Cohort-Study (SMSC): A Prospective Swiss Wide Investigation of Key Phases in Disease Evolution and New Treatment Options. PLoS ONE.

[48] Sakai S., Anada T., Tsuchiya K., Yamazaki H., Margolis H.C., Suzuki O. (2016). Comparative study on the resorbability and dissolution behavior of octacalcium phosphate, β-tricalcium phosphate, and hydroxyapatite under physiological conditions. Dent. Mater. J..

[49] Habibovic P., Sees T.M., van den Doel M.A., van Blitterswijk C.A., de Groot K. (2006). Osteoinduction by biomaterials--physicochemical and structural influences. J. Biomed. Mater. Res. Part A.

[50] Yokoi T., Kim I.Y., Ohtsuki C. (2012). Mineralization of calcium phosphate on octacalcium phosphate in a solution mimicking in vivo conditions. Phosphorus Res. Bull..

[51] Yassine I., Joudi M., Hafdi H., Hayimi B., Moudlar J., Bensemlai M., Nasrellah H., Abderrahim M., Bakasse M. (2022). Synthesis of Brushite from Phophogypsum Industrial Waste. Biointerface Res. Appl. Chem..

[52] Miller M.A., Kendall M.R., Jain M.K., Larson P.R., Madden A.S., Tas A.C. (2012). Testing of Brushite (CaHPO_4_ 2H_2_O) in synthetic Biomineralization and In Situ Crystallization of Brushite Micro-Granules. Am. Ceram. Soc..

[53] Kajiyama S., Yokoi T., Sakamoto T., Ohtsuki T., Inoue M., Kato T., Nishimura T. (2016). Rapid and topotactic transformation from octacalcium phosphate to hydroxyapatite (HAP): A new approach to self-organization of freestanding thin-film HAP-based nanohybrids. Cryst. Eng. Comm..

[54] Asadollahzadeh M., Rabiee S.M., Kenari H.S. (2019). In vitro apatite formation of calcium phosphate composite synthesized from fish bone. Int. J. Appl. Ceram. Technol..

[55] Manafi S.A., Yazdani B., Rahimiopour M.R., Sadrnezhaad S.K., Amin M.H., Razari M. (2008). Synthesis of nano-hydroxiapatite under a sonochemical/hydrothermal condiction. Biomed. Mater..

[56] Dorozhkin S.V. (2017). Calcium orthophosphates (CaPO_4_): Occurrence and properties. Morphologie.

[57] Wang L., Nancollas G.H. (2008). Calcium Orthophosphates: Crystallization and Dissolution. Chem. Rev..

[58] Suzuki O., Shiwaku Y., Hamai R. (2020). Octacalcium phosphate bone substitute materials: Comparison between properties of biomaterials and other calcium phosphate materials. Dent. Mater. J..

[59] Tas A.C. (2011). Granules of Brushite and Octacalcium Phosphate from Marble. J. Am. Ceram. Soc..

[60] Anee T., Sundaram N.M., Arivuoli D., Ramasamy P., Kalkura S.N. (2005). Influence of an organic and inorganic additive on the crystallization of dicalcium phosphate dihydrate. J. Cryst. Growth.

[61] Moseke C., Bayer C., Vorndran E., Barralet J.E., Groll J., Gbureck U. (2012). Low temperature fabrication of spherical brushite by cement paste emulsion. J. Mater. Sci. Mater. Med..

[62] Lu B.-Q., Willhammar T., Sun B.-B., Hedin N., Gale J.D., Gebauer D. (2020). Introducing the crystalline phase of dicalcium phosphate monohydrate. Nat. Commun..

[63] Cama G., Gharibi B., Saif Sait M., Knowles J.C., Lagazzo A., Romeed S., Di Silvo L., Deb S. (2013). A novel method of forming micro- and macroporous monetite cements. J. Mater. Chem. B.

[64] Wang X., Zhang H., Yu X., Mo X., Gao J., Hu Y., Min J., Ding Q., Fan Y., Jiang W. (2024). Effects of water on cold-sintered highly dense dicalcium phosphate anhydrous bioceramic using its hydrate. J. Am. Ceram. Soc..

[65] Alexopoulou M., Mystiridou E., Mouzakis D., Zaoutsos S., Fatouros D.G., Bouropoulos N. (2016). Preparation, characterization and in vitro assessment of ibuprofen loaded calcium phosphate/gypsum bone cements. Cryst. Res. Technol..

[66] Kamieniak J., Banks C.E., Bernalte E., Foster C.W., Doyle A.M., Kelly P.J. (2016). High Yield Synthesis of Hydroxyapatite (HAP) and Palladium Doped HAP via a Wet Chemical Synthetic Route. Catalysts.

[67] Bish D.L., Post J.E. (1993). Quantitative mineralogical analysis using the Rietvetd full-pattern fitting method. Am. Mineral..

[68] Østereng Halvorsen J., Stacey P., Graff P., Folven Gjengedal E.L., Ervik T.K. (2025). Application of X-ray diffraction with Rietveld refinement to quantify mineral composition including crystalline silica in respirable dust. J. Occup. Environ. Hyg..

[69] He W., Xue B., Qian Q., Chen S., Fu Z., Wang K. (2024). Mineralization of Octacalcium Phosphate under Magnetic Field. Crystals.

[70] Zhao X., Jiang S., Rao J., Zhou J., Li Z., Yang J., Yan K., Shi H. (2022). Physicochemical and cytological properties of silicon-doping octacalcium phosphate. Mater. Lett..

[71] (2014). Implants for Surgery: In Vitro Evaluation for Apatite-Forming Ability of Implant Materials (3rd ed.).

[72] Tian B., Chen W., Dong Y., Marymont J.V., Lei Y., Ke Q., Guo Y., Zhu Z. (2016). Silver Nanoparticle-Loaded Hydroxyapatite Coating: Structure, Antibacterial Properties, and Capacity for Osteogenic Induction In Vitro. RSC Adv..

[73] (2019). Standard Guide for Quantitative Analysis by Energy-Dispersive Spectroscopy.

