# Supplementary material for: Morphology-Dependent Transformation of Dicalcium Phosphate Dihydrate (DCPD) to Octacalcium Phosphate (OCP) and Its Stability in Simulated Physiological Fluids
Source: Molecules. 2025 Sep 5;30(17):3631. doi: 10.3390/molecules30173631 (PMC12430153; doi:10.3390/molecules30173631)
Supplement: Supplementary file 1 [file molecules-3788429-supplementary.pdf › molecules-3788429-supplementary.pdf]

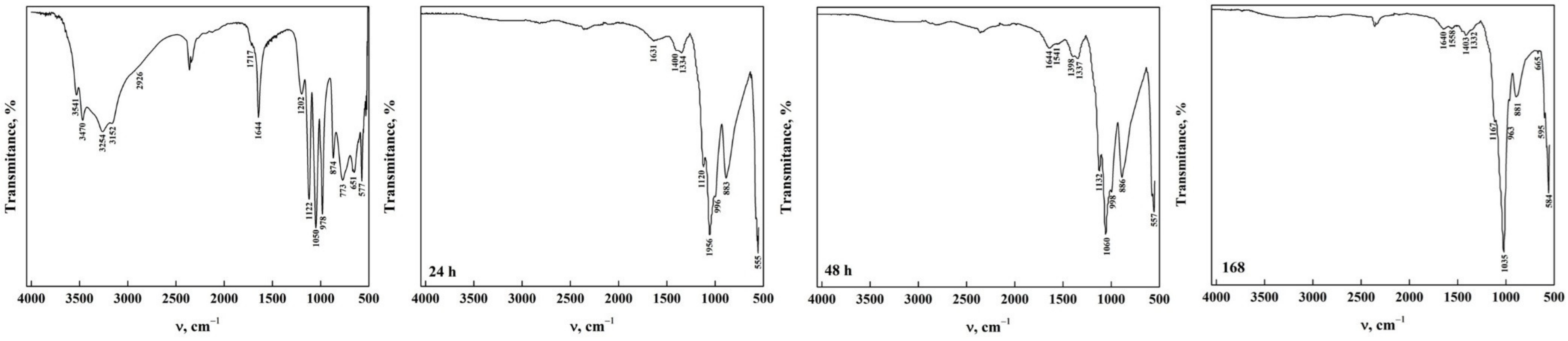

Figure S1. ATR-FTIR spectra of the transformation of DCPD-FP to OCP-FP

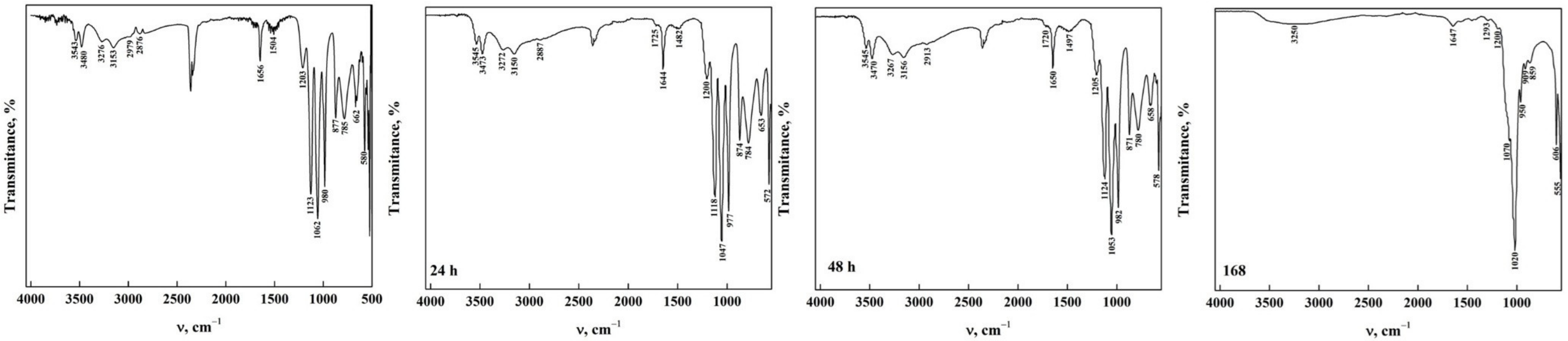

Figure S2. ATR-FTIR spectra of the transformation of DCPD-WL to OCP-WL
